# Complement System as a New Target for Hematopoietic Stem Cell Transplantation-Related Thrombotic Microangiopathy

**DOI:** 10.3390/ph15070845

**Published:** 2022-07-09

**Authors:** Gianluigi Ardissino, Valentina Capone, Silvana Tedeschi, Luigi Porcaro, Massimo Cugno

**Affiliations:** 1 Center for HUS Prevention, Control and Management at Pediatric Nephrology, Dialysis and Transplant Unit, Fondazione IRCCS Ca’ Granda, Ospedale Maggiore Policlinico, 20122 Milano, Italy; ardissino@centroseu.org (G.A.); valentina.capone@policlinico.mi.it (V.C.); 2 Medical Genetics Laboratory, Fondazione IRCCS Ca’ Granda, Ospedale Maggiore Policlinico, 20122 Milano, Italy; silvanatedeschi52@gmail.com (S.T.); luigi.porcaro@policlinico.mi.it (L.P.); 3 Medicina Interna, Dipartimento di Fisiopatologia Medico-Chirurgica e dei Trapianti, Fondazione IRCCS Ca’ Granda, Ospedale Maggiore Policlinico, Università Degli Studi di Milano, Via Pace, 9, 20122 Milano, Italy

**Keywords:** hematopoietic stem cell transplantation, thrombotic microangiopathy, complement, eculizumab, narsoplimab

## Abstract

Thrombotic microangiopathy (TMA) is a complication that may occur after autologous or allogeneic hematopoietic stem cell transplantation (HSCT) and is conventionally called transplant-associated thrombotic microangiopathy (TA-TMA). Despite the many efforts made to understand the mechanisms of TA-TMA, its pathogenesis is largely unknown, its diagnosis is challenging and the case-fatality rate remains high. The hallmarks of TA-TMA, as for any TMA, are platelet consumption, hemolysis, and organ dysfunction, particularly the kidney, leading also to hypertension. However, coexisting complications, such as infections and/or immune-mediated injury and/or drug toxicity, together with the heterogeneity of diagnostic criteria, render the diagnosis difficult. During the last 10 years, evidence has been provided on the involvement of the complement system in the pathophysiology of TA-TMA, supported by functional, genetic, and therapeutic data. Complement dysregulation is believed to collaborate with other proinflammatory and procoagulant factors to cause endothelial injury and consequent microvascular thrombosis and tissue damage. However, data on complement activation in TA-TMA are not sufficient to support a systematic use of complement inhibition therapy in all patients. Thus, it seems reasonable to propose complement inhibition therapy only to those patients exhibiting a clear complement activation according to the available biomarkers. Several agents are now available to inhibit complement activity: two drugs have been successfully used in TA-TMA, particularly in pediatric cases (eculizumab and narsoplimab) and others are at different stages of development (ravulizumab, coversin, pegcetacoplan, crovalimab, avacopan, iptacopan, danicopan, BCX9930, and AMY-101).

## 1. Introduction

The thrombotic microangiopathy (TMA) that complicates both autologous and allogeneic hematopoietic stem cell transplantation (HSCT) is conventionally called transplant-associated thrombotic microangiopathy (TA-TMA). Despite its severity and the many efforts to understand it, the pathogenetic mechanisms still remain largely unknown and its diagnosis can be challenging. The hallmarks of the TA-TMA, as for any TMA, are platelet consumption, hemolysis, and organ dysfunction, particularly the kidney, leading also to hypertension [1]. However, in patients who recently received HSCT, the listed signs can overlap with other conditions and/or may be difficult to identify. For this reason, the epidemiology of TA-TMA remains poorly defined: its frequency ranges from as low as 3% to as high as 39% and the related case-fatality rate has been reported as high as 84%, depending on the definition criteria [2,3,4].

The gold standard for the diagnosis of TA-TMA is based on histologic findings; however, bleeding risks often precludes this type of workout. Several attempts to refine and standardize the definition of TA-TMA on the clinical ground have been made, but a certain degree of heterogeneity in the diagnostic criteria still remains [5,6,7,8,9,10]. Table 1 shows the different criteria used and their development over time responsible for some of the epidemiological differences reported. As mentioned, the heterogeneous diagnostic criteria are basically due to the lack of specific biomarkers as well as the difficulty in the diagnosis for the peculiar circumstances when it usually develops: early in the post-transplant period. During this critical period, patients often exhibit cytopenia with already ongoing low platelet count; thus, platelet consumption often needs to be inferred from indirect, ill-defined evidence, such as the persistence of low platelets and/or the increased transfusion requirement. Most of the indicators of hemolysis (anemia, increased LDH, haptoglobin, schistocytes), as well as those of organ damage (including renal function and blood pressure), may be affected by frequently coexisting complications (infections and/or immune-mediated injury and/or drug toxicity) [11].

Moreover, compared to primary TMAs, the one complicating HSCT is more subtle in its clinical course compared with other primary TMAs, possibly because the coexisting low platelet count may reduce the formation of thrombi on damaged endothelium, thus reducing the severity of microvascular occlusion.

TA-TMA is considered a syndrome of abnormal endothelial cell activation and injury [12,13]. Various factors in the transplant process may lead to the development of endothelitis, subsequent formation of platelet-rich thrombi, and the described end-organ dysfunction. A three-hit hypothesis has been proposed and widely accepted: (1) an underlying predisposition to complement activation or pre-existing endothelial injury (hit 1); (2) pre-existing toxicity by the conditioning regimen (hit 2); (3) additional insults, including medications, alloreactivity, and/or infections (hit 3) [14]. The endothelial injury leads to an increase in pro-inflammatory cytokines and procoagulant factors that further promote tissue damage.

Well recognized risk factors for TA-TMA are female gender, prior transplant, primary disease, mismatched or unrelated donor, myeloablative conditioning regimen, graft-versus-host disease (GVHD), and its prophylaxis with sirolimus and/or calcineurin inhibitors (CNIs) and pre-transplant kidney dysfunction [9].

Recently, studies have concentrated on the possible role of complement in the pathogenesis of TA-TMA, in particular the role of genetic predisposition to abnormal activation of the complement cascade. The present paper is aimed to retrace the possible role of complement in the pathogenesis of TA-TMA by exploring the available evidences on the genetic and functional aspects as well as on the therapeutic ground.

## 2. Literature Search

The following keywords were used to identify relevant studies published in PubMed before May 2022: “hemolytic uremic syndrome,” “HUS,” “thrombotic microangiopathy,” and “TMA.” We then excluded all the papers that did not deal with “hematopoietic stem cell transplantation,” “bone marrow transplantation,” or “HSCT.” Moreover, papers dealing with “thrombotic thrombocytopenic purpura” or “TTP” were excluded. The search was limited to English-language publications only.

## 3. Complement System Involvement

**Figure 1:**
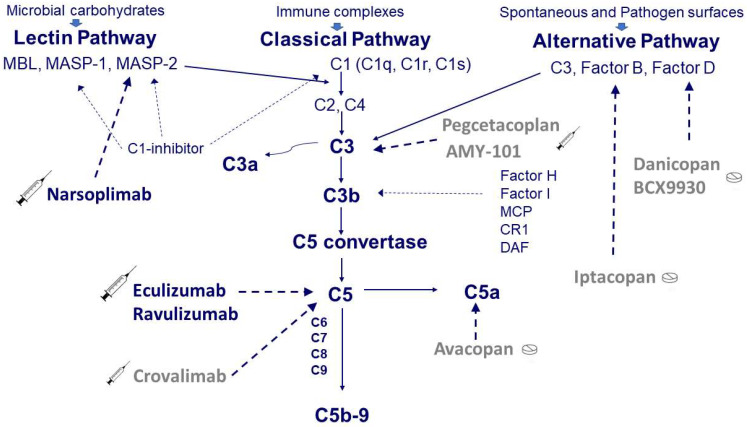
Simplified scheme of the complement system and target sites of the available drugs. Complement can be activated through three pathways: the classical pathway triggered by antibody-antigen complex, the alternative pathway spontaneously activated at a low level or triggered by specific surface antigens and the lectin pathway activated by binding mannose residues on the pathogen surface. The classical pathway starts from the three components of C1, i.e., C1q and the two proteases C1r and C1s. The activation of C1 in turn induces the activation of C2 and C4, which are also activated by the proteases associated with the mannose-binding lectin (MBL), i.e., MASP-1 and MASP-2. The activation of the classical and lectin pathways is controlled by a C1-inhibitor that can block C1r, C1s, MASP-1, and MASP-2. The alternative pathway, composed of C3, Factor B, and Factor D, is regulated by soluble inhibitors such as factor H and factor I as well as by cell-bound inhibitors such as membrane cofactor protein (MCP), complement receptor 1 (CR1), and decay-accelerating factor (DAF). The activation of the three pathways (classical, lectin, and alternative) converges on the common pathway with the formation of strong inflammatory mediators, such as C3a and C5a, and the production of the C5b-9 membrane attack complex (MAC) that lyses target cells. Therapy with eculizumab blocks C5, whereas therapy with narsoplimab blocks MASP-2. Although never used in TA-TMA, other complement inhibitory drugs are available at different stages of development, such as crovalimab for C5, iptacopan for factor B, danicopan for factor D, and pegcetacoplan for C3.

The complement system is an important mediator of the innate immune response and represents one of the main defense mechanisms against infectious agents [15,16]. Moreover, it is also involved in the clearance of self-antigens derived from apoptotic processes and tissue repair [16]. The system consists of a large number of proteins that are present in circulating blood and tissues. These proteins work in a coordinated manner (Figure 1) towards the activation of three pathways: the classical pathway triggered by antibody-antigen complex, the alternative pathway triggered by specific surface antigens, and the lectin pathway by binding mannose residues on the pathogen surface. The alternative pathway is in a constant state of activation at a low level, known as ‘tickover,’ allowing a prompt response upon microorganism challenge. The three pathways converge on the common pathway with the formation of strong inflammatory mediators, such as C3a and C5a, and the production of the C5b-9 membrane attack complex (MAC) that lyses target cells [15,16]. The system is tightly regulated by soluble inhibitors such as C1-inhibitor, factor H, and factor I as well as by cell-bound inhibitors such as membrane cofactor protein (MCP), complement receptor 1 (CR1), and decay-accelerating factor (DAF) [15]. Being an important part of the immuno-defense system, the complement system plays a critical role in concert with coagulation in promoting the inflammatory process that may also lead to tissue injury if overactivated [16]. Although the interactions between complement and coagulation and their role in inflammation are well known [17], to the best of our knowledge, these relationships are not completely clear in the thrombotic microangiopathies complicating the transplants of hemopoietic stem cells (TA-TMA).

### 3.1. Functional Aspects

In 1975, based on experimental evidence obtained in a GVHD rat model, complement activation was first suspected to be responsible for some complications observed in patients with bone marrow transplants [18]. In the same period, complement activation was described in 4 patients with the TMA of the hemolytic uremic syndrome (HUS) [19] and in the subsequent years, complement activation has become the hallmark of atypical HUS (the HUS not associated with shigatoxin-producing *E. coli* infections) [20], paving the way for the successful treatment of the disease with complement-blocking drugs [21]. In contrast, a clear association between complement activation and TA-TMA has not been demonstrated so far, though several reports on this association are present in the literature of the last 10 years, and several attempts have been made to identify the patients with TA-TMAs that might respond to complement blocking therapy. To achieve this purpose, various problems still need to be solved. Although among the many complications of HSCT, the first report of TMA appeared in the late 1980s [22], after more than 30 years, a univocal clinical characterization of TA-TMA is still lacking and to date, various criteria have been proposed, as reported above [5,6,7,8,9,10].

The first evidence of complement activation in TA-TMA were provided in 2011 by Mii et al. [23] and in 2013 by Laskin et al. [24], showing the glomerular deposition of C4d, a well-known marker of classic complement activation. Mii et al. described a diffuse C4d deposition in glomerular capillaries in 2 biopsy and 2 autopsy cases out of 7 cases of TA-TMA [23]. Laskin et al. studied 20 pediatric HSCT recipients, of whom 8 developed TA-TMA, and found that diffuse or focal renal arteriolar C4d staining was more common in subjects with histologic TA-TMA (75%) compared with patients who did not develop TA-TMA (8%) [24]. Thus, both studies demonstrated that in more than 50% of patients with TA-TMA diagnosis, confirmed by histological evaluation, there is complement activation possibly playing a role in renal tissue injury. In 2014, Jodele et al. found elevated levels of soluble terminal complement complex (sC5b-9) in plasma from 5 of 6 children with TA-TMA, and 4 of them were successfully treated by blocking terminal complement with eculizumab [25]. In 2017, increased plasma levels of sC5b-9 were also found by Qi et al. in 20 patients with TA-TMA compared with 20 patients who did not present complications during the post-transplant follow-up [26]. In 2020, Mezo et al. demonstrated the association between the early increase in sC5b-9 in plasma and the development of TA-TMA during the first 100 days post-transplantation (10 of 10 pediatric patients with TA-TMA vs. 27 of 57 without) [27]. In 2020, Gavriilaki et al. found that sC5b-9 plasma levels were significantly higher in 20 adult patients with TA-TMA compared with 20 patients with GVHD and 20 control patients (*p* < 0.001). Moreover, an association between sC5b-9 plasma levels and an index of endothelial activation/injury was also reported [28]. Moreover, Okamura et al. found a significant predictive value of early (at 7 days after transplant) high plasma levels of the Ba, a fragment released from complement factor B upon activation of the complement alternative pathway [29].

### 3.2. Genetic Aspects

Among the mechanisms responsible for complement activation in TA-TMA, the structural and point alterations in the cluster of complement regulatory genes (CFH, CFB, CFI, C3, CD46/MCP, DGKE, THBD, CFHR) are the ones mostly accepted today. In 2013, following the similarities between TA-TMA and atypical hemolytic uremic syndrome (aHUS), Jodele et al. first suspected the presence of genetic variants in the complement Factor H (CFH) through the identification of deletions in CFH-related genes 3 and 1 (delCFHR3-CFHR1) in 5 out of 6 children with TA-TMA and IgG autoantibodies against FH in three [30]. Autoantibodies have also been found by our group in 7 of 20 TA-TMA patients: in 4 patients, the autoantibodies were of the IgG class, whereas in 3 were of the IgM class; the latter were not associated with CFHR3-CFHR1 deletion [31]. Genetic alterations in TA-TMA patients were confirmed in a large prospective investigation by Jodele et al. [32]. In this study, pre-transplant genomic DNA was analyzed in 77 pediatric patients undergoing HSCT (of whom 34 developed TA-TMA) for 17 genes involved in the regulation of the complement pathways. Gene variants were detected in 65% of TA-TMA patients (regardless of race) compared with 9% of patients without TMA [32]. Multiple variants (rare, with minor allele frequency < 1%, or of uncertain significance [VUS] or likely benign) were common in African Americans and were associated with a higher case-fatality rate (71%), suggesting a cumulative effect of the detected complement gene variants [32]. These results first introduced the concept of a possible genetic susceptibility to TA-TMA based on the recipient’s genotype leading to complement upregulation even in the presence of genetic variants with little or no functional significance [32]. In 2020, Gavriilaki confirmed the same findings in 40 TA-TMA adult patients showing a significantly higher frequency of pathogenic and rare variants [33]. Furthermore, most of the variants located in exonic/splicing/untranslated regions of complement-related genes and of ADAMTS13 were associated with poor outcomes. The finding of ADAMTS13 gene variants seems to be consistent with the experimental results obtained by Zheng et al. in a mouse model of TMA, which supported a synergistic effect of ADAMTS13 deficiency and complement activation in the pathogenesis of TMA [34]. However, the importance of complement-related genetic variants in TA-TMA was denied by Okamura et al. [29]. In their nested case-control study of 15 TA-TMA patients and 15 non-TA-TMA patients, no significant differences were identified between the two groups as far as variants in 17 complement system-related genes. Nevertheless, according to the authors, early high levels of the complement activation biomarker Ba are predictive of TA-TMA [29].

In 2017, our group [35] reported complement gene variants in 6 of 16 DNA samples from transplanted patients with full chimerism (donor’s DNA) in the absence of variants in the pretransplant patient’s DNA (recipient’s DNA) analyzed by targeted Next Generation Sequencing (NGS) and Multiplex ligation-dependent probe amplification (MLPA) analysis. We suggested that transplanted monocyte-derived cells, if mutated, may be responsible for the production of abnormal complement regulatory proteins, leading to complement dysregulation and TA-TMA. This may occur due to colonization of the recipient’s liver by donor-derived hematopoietic stem cells carrying variants, with the consequent transfer of the genetic risk. Thus, the two hit hypotheses of aHUS may also apply to TA-TMA (these may represent 2 of the 3 hits previously mentioned) to the extent that donors carrying variants do not exhibit the disease in the absence of complement activating triggers, well present in recipients after HSCT. These data suggest that a pre-transplantation genetic screening of both recipients and donors may provide additional data on the pathophysiology of TA-TMA. The possible contribution of donor-derived genetic risk factors was further strengthened by Rodrigues et al. in 2021 with a larger analysis of 33 TA-TMA patients [36], in which both pretransplant and donor’s DNAs were investigated by complement genes targeted NGS and MLPA. The number of patients with variants in donor DNA was significantly greater than that of patients with variants in pre-transplant DNA, 33% compared to 12%, respectively, with multiple variants detected in 5 donor DNAs. However, Gavriilaki et al. [33] have challenged the notion that a donor’s DNA may play a role in the development of TA-TMA. In this study, significantly lower frequencies of pathogenic and rare variants in the cluster of complement regulatory genes were detected in donors and controls compared to pretransplant TA-TMA recipients that showed several pathogenic or likely pathogenic variants.

At the last American Society of Hematology meeting, Zhang et al. presented preliminary data obtained by Whole Exome Sequencing (WES) in pre-transplant DNA, focusing the research on 5 genetic pathways: complement regulation (17 genes), VWF and coagulation (7 genes), VWF clearance (10 genes), ADAMTS13 mimics or interacting proteins (10 genes), and angiopoietin family and endothelial activation (7 genes). They did not show differences in the presence of variants in complement-related genes between 91 patients who developed TA-TMA and 93 who did not. A significant association was identified only in the VWF clearance pathway. Rare variants in the LRP1 gene coding for a member of the low-density lipoprotein receptor family were found but with no predicted pathogenicity. Impaired VWF clearance is associated with a predisposition for complement activation as the binding of VWF to complement proteins activates the complement system and this mechanism has been observed in TA-TMA patients [37].

The discrepancy between findings from different authors regarding variants in the alternative pathway of complement (Table 2) may be explained by a different approach to variant classification. In fact, the absence of functional studies for many of the identified variants entails the use of bioinformatics tools to predict their possible pathogenicity. The predictions are often contradictory, probably depending on the chosen reference transcripts and/or the use of different algorithms with consequent variability in variant annotations. This may generate conflicting conclusions on the real functional effect of the nucleotide changes. Moreover, it should be acknowledged that most of the studies are underpowered due to the limited number of patients.

Nowadays, the basic molecular diagnostics in TA-TMA patients should include genetic screening for variants, polymorphisms, at-risk haplotypes (CFH-H3; MCPggaac), and macrorearrangements (CFH/CFHR hybrid genes) in the previously mentioned complement genes cluster. Given the contradictory findings on the role of complement gene abnormality that alone cannot fully explain the pathogenesis of TA-TMA, we believe that investigations should be extended beyond complement by including its interaction with inflammatory, coagulation, and other pathways involved in endothelial injury, as recently suggested by Jodele et al. [38]. Moreover, for the same conflicting results on the role of complement-related gene abnormalities in patients and donors, further investigation is needed before a pre-transplant genetic screening can be proposed for the evaluation of TA-TMA risk.

### 3.3. Therapeutic Aspects

Following the increasing evidence of the impressive efficacy of complement inhibition in the treatment of aHUS [39] and given the mentioned similarities between the latter disease and TA-TMA, since 2011 (the year of eculizumab approval for aHUS by the FDA), there has been a growing interest in the role of complement as a potential target to improve the outcome of this severe complication of HSCT. During the previous two decades, therapeutic strategies for TA-TMA have been poorly standardized and encompassed conventional approaches, including withdrawal of drugs potentially causing endothelial damage (such as calcineurin-inhibitors and mTOR inhibitors), plasma exchange, rituximab, defibrotide, and daclizumab [40,41]. The withdrawal (or the dose reduction) of calcineurin-inhibitors and mTOR inhibitors remains a standard of care in the management of TA-TMA; however, it should be considered that immunosuppression tapering may exacerbate GVHD and thus even trigger TA-TMA itself [42]. Although some initial reports have shown encouraging results with the use of plasma exchange in this setting, the overall efficacy did not stand the test of time [43,44]. Rituximab, a chimeric monoclonal antibody directed toward CD20 antigen, whose exact mechanism of action in TA-TMA is all but clear, showed evidence of response but based on single patient reports [30,45,46]. Defibrotide, a mixture of single-stranded oligonucleotides purified from the intestinal mucosa of pigs, with a protective effect on the endothelium as well as local antithrombotic and fibrinolytic action, showed a promising effect in need of confirmation with prospective trials [47,48]. The replacement of calcineurin-inhibitors or mTOR inhibitors with interleukin-2 inhibitors has shown encouraging results [49].

As mentioned above, from 2011, the interest has shifted and concentrated on complement blockade. Figure 1 shows the sites of action of complement inhibitory drugs presently available. Some have already been used in TA-TMA, while others may represent a future option (Table 3).

Eculizumab (Soliris, Alexion Pharmaceuticals), a terminal complement inhibitor, is a humanized monoclonal antibody that binds with high affinity to the human C5 complement protein and blocks the generation of proinflammatory C5a and C5b-9. It is approved for the treatment of paroxysmal nocturnal hemoglobinuria [62] and atypical hemolytic uremic syndrome [63]. The drug is administered intravenously and has a half-life of 12.5 days. The standard induction schedule in adults for the indication of aHUS consists of 5 consecutive doses at a one-week interval (900 mg in the initial 4 doses and 1200 mg in the 5th one), while in children, the schedule is adjusted according to body weight. The induction treatment is followed by a maintenance schedule consisting of 1200 mg every two weeks life-long. Several pieces of evidence indicate that the maintenance schedule can be individualized by monitoring complement activity and the interval safely and effectively extended to 3 or 4 weeks [64,65,66] and in a few cases even to 5 weeks. Although the drug has been registered for life-long use, discussions are ongoing on the possibility of discontinuing C5 inhibition once the disease is in stable remission, particularly in cases without associated complement abnormality [50,67].

Eculizumab was used for the first time in TA-TMA in 2013, as reported by Peffault de Latour et al. [68], who successfully treated a 61-year-old man with multiple myeloma developing TA-TMA after a tandem autologous-allogeneic HSCT. However, most literature on the use of eculizumab in TA-TMA derives from pediatric cohorts with several small case series and promising but insufficiently conclusive results [25,51,52]. In particular, in 2020, Jodele et al. described the effect of eculizumab in a prospective study including a subgroup of 64 out of 177 children (<18 years of age) with high-risk TA-TMA [51]. The subgroup met the following criteria: 1) nephrotic range proteinuria; 2) activated complement system as shown by elevated plasma levels of sC5b-9 (≥244 ng/mL) or one of the two criteria along with either clinical evidence of multi-organ dysfunction or biopsy-proven TMA. Eculizumab dose and interval were adjusted to maintain a serum trough level of ≥100 ug/mL and CH50 fully suppressed (<10%). The study demonstrated a response rate of 64% and a survival rate of 66% 1 year after HSCT, compared with 16.7% survival in a historical untreated control cohort. The observed response rate was lower in patients with higher baseline sC5b-9 levels as well as in patients with delayed initiation of treatment or with massive intestinal bleeding. Indeed, in a previous paper, the same authors showed that bleeding patients with TA-TMA had a faster eculizumab clearance, thus requiring more drug (20 doses vs. 9) and a lower 1-year survival compared with those without bleeding complications (44% vs. 78%) [53]. Based on these observations, Mizuno et al. identified subjects with TA-TMA and clinically significant bleeding as an ultra–high-risk group in need of personalized drug dosing to improve survival [52]. In this study, eculizumab pharmacokinetics and pharmacodynamics were analyzed in 19 bleeding and 38 non-bleeding patients (0.5–29.9 years of age), showing that sC5b-9 and body weight were significant determinants of eculizumab clearance besides bleeding. In detail, eculizumab clearance after the first dose was higher in bleeding than in non-bleeding patients (83.8 vs. 61.3 mL/h per 70 kg of body weight) and the higher clearance was maintained over treatment doses in bleeding patients, whereas non-bleeding patients showed a time-dependent decrease in drug clearance. However, the significant decrease in sC5b-9 levels observed in all patients irrespective of bleeding status raises some concern about the role of pharmacokinetic parameters in this context. In our experience, the management of drug schedule (dose and interval) can be alternatively and more efficiently based only on pharmacodynamic parameters targeted on complement inhibition both in primary aHUS and in TA-TMA [66]. Ibrahimova et al., in a recent abstract [54], showed that elevated thrombin generation, especially in patients with intestinal bleeding, likely contributes to eculizumab refractoriness in 12 TA-TMA patients. Adults cohorts of TA-TMA patients treated with C5 inhibitor are small (5 to 12 patients) and show variable as well as less impressive results: survival ranged between 33 and 60% [55,56,57] and hematologic response between 50 and 93% [55,57,58]. However, a poor impact on kidney recovery has been observed with a high need for chronic kidney replacement therapy [56]. Concerning the duration of C5 inhibition treatment, according to Jodele et al. [51], it can be discontinued when hematologic and renal responses have been achieved. Even in our experience, the treatment with eculizumab can be safely discontinued with an acceptable risk of relapse, particularly in those patients who do not carry any significant complement abnormality. Patients who have discontinued eculizumab treatment can be easily monitored with a urine dipstick for hemoglobinuria, which has been proven to be highly sensitive for the early detection of relapses [59].

Ravulizumab (ALXN1210; Alexion Pharmaceuticals, Inc., Boston, MA, USA) is a new C5 inhibitor that achieves complete and sustained inhibition of complement activity with an extended dosing interval and a higher binding affinity to C5 compared with eculizumab [69,70]. Ravulizumab was designed via targeted substitution of 4 amino acids in the complementary binding and Fc region of the eculizumab structure, resulting in augmented endosomal dissociation of C5 and efficient recycling of ravulizumab to the vascular compartment. Actually, it has been demonstrated that the ability of mAb-C5 complexes to dissociate in the endosomes of cells is a strong determinant of the duration of mAb action in vivo. Therefore, increasing the dissociation in the endosome allows the antibody to partake in additional rounds of C5 binding and neutralization [71]. Accordingly, the terminal half-life of ravulizumab is ~4 times longer than that of eculizumab. As with eculizumab, a >99% reduction in free C5 has been observed as early as the end of the first intravenous infusion of ravulizumab [72].

C5 is also targeted by a novel monoclonal antibody, Crovalimab, currently in a phase III trial in patients with aHUS (NCT04861259), that will allow self-subcutaneous injection and will also be available for patients with resistance to eculizumab, thanks to its binding site on a different C5 epitope [73].

Coversin, another C5 inhibitor, is a small recombinant compound derived from a protein found in the saliva of the *Ornithodoros moubata* tick. This molecule works by damping down the local complement-mediated inflammatory response of the host animal enabling the tick to repeatedly feed without damage from the host. Although coversin is not a monoclonal antibody, it binds to C5 in a different location than eculizumab, but the final effect is the same. Coversin was successfully used in a TA-TMA patient with a C5 variant that caused resistance to eculizumab treatment [74].

Avacopan is an orally-administered inhibitor of the complement C5a receptor successfully used as adjuvant treatment in antineutrophil cytoplasmic antibody (ANCA)-associated vasculitis [60]. It is also included in ongoing clinical trials on C3 glomerulopathy (ClinicalTrials.gov. NCT03301467), hidradenitis suppurativa (NCT03852472), IgA nephropathy (NCT02384317), and atypical hemolytic uremic syndrome (NCT02464891).

New therapeutic approaches encompass narsoplimab (OMS721), a human monoclonal antibody against MASP-2 (Mannose-binding lectin Associated Serine Protease-2), the effector enzyme of the lectin pathway in the complement system. In addition, MASP-2 is an activator of the coagulation cascade via prothrombin cleavage to form thrombin leading to the generation of fibrin [75,76,77]. Narsoplimab, blocking MASP-2, exerts an inhibitory action on both complement and coagulation, and thus it potentially reduces microvascular thrombosis [78]. Narsoplimab has been used in COVID-19 patients [78], showing a reduction of endothelial cell damage. In a single-arm open-label pivotal trial (NCT02222545), in which 28 adult patients with TA-TMA received narsoplimab, a response rate of 61% was observed [79].

Another specific complement inhibitor currently approved for PNH is Pegcetacoplan, which binds to complement protein C3 and to its activation fragment, C3b, regulating C3 cleavage and the downstream activation. A study to evaluate the pharmacokinetics, efficacy, safety, and tolerability of Pegcetacoplan in patients with TA-TMA is ongoing (ClinicalTrials.gov. NCT05148299).

Two additional inhibitors of the alternative pathway, Iptacopan and Danicopan [61], are available. Iptacopan (LNP023) is a new, oral, selective inhibitor of factor B, currently in a phase III trial in patients with aHUS (Efficacy and Safety of Iptacopan [LNP023] in Adult Patients With Atypical Hemolytic Uremic Syndrome Naive to Complement Inhibitor Therapy; ClinicalTrials.gov. NCT04889430). Danicopan is a Factor D inhibitor studied as an add-on therapy to a C5 inhibitor (eculizumab or ravulizumab) in patients with PNH who have clinically evident extravascular hemolysis (Danicopan as Add-on Therapy to a C5 Inhibitor in Paroxysmal Nocturnal Hemoglobinuria (PNH) Participants Who Have Clinically Evident Extravascular Hemolysis; ClinicalTrials.gov. NCT04469465). There is another oral Factor D inhibitor named BCX9930 that is included in ongoing development programs in the treatment of paroxysmal nocturnal hemoglobinuria (ClinicalTrials.gov. NCT04702568, NCT05116787, NCT04330534, NCT05116774) and C3 glomerulopathy (NCT05162066).

Finally, an additional complement inhibitor drug targeting C3 is AMY-101, which has been tested in adults with gingivitis (ClinicalTrials.gov. NCT03694444) and in patients with acute respiratory distress syndrome due to COVID-19 (ClinicalTrials.gov. NCT04395456).

A recent systematic review and meta-analysis including 116 patients from 6 studies suggest that eculizumab is safe in patients with TA-TMA and it also improves recovery and survival rate [80] in both children and adults. To date, the evidence of the clinical efficacy of eculizumab in TA-TMA is mainly based on data from small observational studies. Thus, the scientific community is eager to see the results of the ongoing randomized controlled studies on the long-acting C5 inhibitor ravulizumab in TA-TMA (Ravulizumab in Thrombotic Microangiopathy After Hematopoietic Stem Cell Transplant. ClinicalTrial.gov. NCT04543591).

## 4. Conclusions

Several pieces of evidence (functional, genetic, and therapeutic) point to a possible role of complement in the pathogenesis of TA-TMA; however, they are insufficient to support a systematic use of complement inhibition therapy in all patients. More data are needed to rule in or out the possibility that complement activation is specific and not just the byproduct of other pathogenetic mechanisms. Indeed, the evidence so far available indicates that TA-TMA may well be the final results of several different pathogenetic mechanisms, sometimes coexisting and all leading to endothelial injury. The essential condition to shed light on this matter is to identify reliable, early, and shared diagnostic criteria for TA-TMA. In the meantime, it seems reasonable that those patients exhibiting a clear activation of the complement system according to the available biomarkers (sC5b-9 or Ba) are promptly referred to C5 inhibition therapy. This could be particularly important given the damaging effect of complement hyperactivation and its association with a high risk of fatal outcomes.

## Figures and Tables

**Table 1:** Different diagnostic criteria for transplant-associated thrombotic microangiopathy (TA-TMA) and the corresponding reported frequency.

	CTN [5]	IWG [6]	COH [7]	Cho et al. [8]	Uderzo et al. [9]	Jodele et al. [10]
**De novo thrombocytopenia**	**-**	**yes**	**yes**	**yes**	**yes**	**yes**
**De novo anemia**	**-**	**yes**	**-**	**yes**	**yes**	**yes**
**Schistocytosis**	**yes**	**yes**	**yes**	**yes**	**yes**	**yes**
**Negative Coombs test**	**yes**	**-**	**-**	**yes**	**yes**	**-**
**Decreased haptoglobin**	**-**	**yes**	**-**	**yes**	**-**	**-**
**Increase in LDH**	**yes**	**yes**	**yes**	**yes**	**yes**	**yes**
**Renal or Neuro-dysfunction**	**yes**	**-**	**yes**	**-**	**yes**	**yes**
**Hypertension**	**-**	**-**	**-**	**-**	**yes**	**yes**
**Proteinuria**	**-**	**-**	**-**	**-**	**yes**	**yes**
**Increased sC5b9**	**-**	**-**	**-**	**-**	**yes**	**yes**
**Frequency of TA-TMA (%)**	**-**	50	**17**	**13**	**-**	**18**

CTN: Blood and Marrow Transplant Clinical Trials Network; IWG: International Working Group. The relative reference numbers are reported in parenthesis.

**Table 2:** Evidence of genetic and functional involvement of complement in TA-TMA.

	Genetic Alterations in Complement Regulation (Positive Subjects/Investigated Subjects)		Anti-Factor H Auto-ab (Positive Subjects/Investigated Subjects)	Complement Functional Involvement
	**Receiver’s DNA**	**Donor’s DNA**		
**Mii et al. 2011** [23]	-	-	-	C4d glomerular deposition
**Laskin et al. 2013** [24]	-	-	-	C4d glomerular deposition
**Jodele et al. 2013** [30]	5/6	1/6	3/6	-
**Jodele et al. 2014** [25]	-	-	-	Elevated plasma levels of sC5b-9
**Jodele et al. 2016** [32]	22/34	-	-	-
**Ardissino et al. 2017** [35]	0/16	6/16	-	-
**Qi et al. 2017** [26]	-	-	-	Elevated plasma levels of sC5b-9 and C3b
**Gavriilaki et al. 2020** [28]	-	-	-	Elevated plasma levels of sC5b-9
**Gavriilaki et al. 2020** [33]	31 */40	5 */18	-	Elevated plasma levels of sC5b-9
**Mezo et al. 2020** [27]	-	-	-	Elevated plasma levels of sC5b-9
**Cugno et al. 2021** [31]	-	4/20	7/20	-
**Rodriguez et al. 2021** [36]	4/33	11/33	-	-
**Okamura et al. 2021** [29]	9/15	-	-	Elevated plasma levels of Ba
**Zhang et al. 2021** [37]	9 */91	-	-	-

* number of identified variants.

**Table 3:** Complement targeting drugs used in transplant-associated thrombotic microangiopathy (TA-TMA) or potentially useful.

Drug	Type	Mechanism of Action	Mode of Administration	Response Rate	References
	Used in TA-TMA				
Eculizumab	mAb	Inhibition of C5 and C5b9 formation	intravenous	Children 67–78%Adults 33–60%	[25,50,51,52,53,54,55,56,57,58,59]
Ravulizumab	mAb	Inhibition of C5 and C5b9 formation	intravenous	Under evaluation	NCT04543591
Coversin	Small protein	Inhibition of C5 and C5b9 formation	subcutaneous	Single case	[74]
Narsoplimab	mAb	MASP-2 inhibition	intravenous	Adults 61%	[79] NCT02222545
Pegcetacoplan	Peptide	C3 inhibition	subcutaneous	Under evaluation	NCT05148299
	Never used in TA-TMA				
Crovalimab	mAb	Inhibition of C5 and C5b9 formation	subcutaneous		
Iptacopam	Small molecule	Factor B inhibition	oral		
Danicopan	Small molecule	Factor D inhibition	oral		
BCX9930	Small molecule	Factor D inhibition	oral		
Avacopan	Small molecule	C5a inhibition	oral		
AMY-101	Peptide	C3 inhibition	subcutaneous		

## Data Availability

Data sharing not applicable.
